# Farmer and market vendor perceptions of COVID-19 impacts on horticultural fresh food systems in Tonga, Fiji, and Samoa

**DOI:** 10.1186/s40066-023-00406-8

**Published:** 2023-03-03

**Authors:** Steven J. R. Underhill, Soane Patolo, Seeseei Molimau-Samasoni, Salesh Kumar, Sarah Burkhart

**Affiliations:** 1 Australian Centre for Pacific Islands Research, University of the Sunshine Coast, Sippy Downs, Queensland, Australia; 2 Mainstreaming of Rural Development Innovation (MORDI) Tonga Trust, Nuku’alofa, Tonga; 3 Scientific Research Organisation of Samoa, Apia, Samoa; 4 College of Agriculture, Fisheries and Forestry, Fiji National University, Koronivia, Fiji Islands

**Keywords:** COVID-19, Pacific, Small-island developing states, Horticulture, Postharvest, Smallholder farmer

## Abstract

**Background:**

When the global COVID-19 pandemic and state of emergency was declared in early 2020, South Pacific Island nations rapidly closed their borders resulting in significant socio-economic upheaval. With the South Pacific region highly vulnerable to external shocks, there was concern amongst Pacific governments and international donors as to the implications of COVID-19 restrictions on the local food system.

**Methods:**

Horticultural farmers and market vendors (*n* = 825) were surveyed in Fiji, Tonga, and Samoa, using local enumerators, over a five-month period (July to November 2020), which represented the initial phase of COVID-19 restrictions in the region. Data were disaggregated based on location, farmer and vendor impacts, and postharvest loss.

**Results:**

Farmers in Fiji (86%) were more likely to experience difficulties in selling their crops during the initial stages of COVID-19 restrictions, compared to farmers on the smaller Pacific Island nations of Tonga (10%) or Samoa (53%). While market vendors in Fiji (73.2%) and Tonga (56.8%) were similarly impacted, few vendors (22%) in Samoa were affected. Farmers and market vendors on the islands of Viti Levu (Fiji) and Upolu (Samoa), specifically those supplying or located in the key urban centres were more likely to experience elevated postharvest loss. Elevated postharvest loss due to COVID-19 was more prevalent amongst municipal market vendors, peri-urban farms and vendors sourcing from larger commercial farms. Road-side vendors and vendors in the rural areas were less likely to incur elevated loss.

**Conclusions:**

While fresh horticultural food systems in Fiji, Tonga, and Samoa were all adversely effected by COVID-19 restrictions, these impacts were more acute in Fiji. Given value chains associated with main urban centres were more likely to incur elevated postharvest loss, this would imply consumers were avoiding town centres and alternatively sourcing fresh fruit and vegetable from rural road-side vendors. Pacific road-side vendors appear to have provided an important fresh food distribution capacity during local COVID-19 travel restrictions.

## Introduction

With the World Health Organisation (WHO) declaration of a global pandemic in March 2020, a state of emergency and international border closures effectively isolated the Pacific from the rest of the world. The international tourism industry in the Pacific, a pivotal part of many Pacific Island economies effectively ceased overnight [1–4]. Local travel restrictions isolated whole communities and closed non-essential businesses [5], leading to extensive urban unemployment [3, 6]. International COVID-19 responses created wider structural impacts, disrupting trade flow [2, 3, 7], reduced remittance payments [8, 9], limited food imports, and isolated seasonal workers [8]. In Fiji, the initial phase of the pandemic also coincided with category 5 Tropical Cyclone Harold, further compromising local COVID-19 response capacities [5, 10].

Given Pacific horticultural food systems are highly vulnerable to external shocks [8, 11, 12], there was justifiable concern amongst Pacific governments and international donors as to the implications of COVID-19 on the local fresh food system.

Recent studies provide some initial insight into the early impacts of COVID-19 on the fresh food system in the Pacific [2, 10, 13–15]. Government COVID-19 policies and high unemployment resulted in rapid de-urbanisation with population drift back to customary land, particularly in parts of Fiji, Solomon Islands, PNG, and Timor-Leste [2, 10]. The resultant influx into rural communities led to land access disputes, increased larceny, critical farm-input constraints (water, seed, planting material, fertiliser, and farm equipment), and concerns over land clearing and unstainable land use [3, 10].

Pacific government interventions to support the fresh food system initially focused on increasing domestic horticultural production. Smallholder farmers were supported to plant fast-yielding food crops, while peri-urban and transient urban communities unable to relocate to customary land were encouraged to adopt home gardening [10, 16]. In Fiji, the resultant increase in domestic commercial horticultural supply, contrasted with declining local demand due to alternative sourcing from home gardens and household trade, restrictions on social and community gatherings, and reduced access to public transport [10, 14, 16, 17]. Commercial farmers with surplus product due to a cessation of export and tourism-based markets re-directed supply into the local markets. Resultant market over-supply led to price discounting [14], high levels of postharvest loss [8], and declining farmer and vendor market participation [10].

Temporary closures or reduced trading hours in the main municipal fruit and vegetable markets added further complexities. In Fiji, market closures restricted supply and led to sporadic price rises [16]. In PNG, there was a reduction in fresh food supply and declining vendor participation in urban areas [3]. In the Solomon Islands, there was declining participation in commercial agricultural food supply [10].

Collectively, COVID-19 impacts on commercial horticultural fresh foods systems in the Pacific appear to have been highly incongruent, fluctuating between periods of market over-supply and reduced food accessibility. Urban households more reliant on commercially sourced food appear to have been particularly vulnerable to this market supply volatility [8, 10].

While the full impact of COVID-19 on Pacific food systems is still evolving, relatively little has been reported on the possible impacts amongst the smaller Pacific Island nations. In this study, we assessed the potential impact of COVID-19 on horticultural farmers and market vendors in Tonga and Samoa, two relatively small Pacific Island nations, as well as in the larger Fiji Islands. Particular attention is given to possible elevated farm and market vendor horticultural food loss due to COVID-19 restrictions.

## Methods

### Study area

**Fig. 1:**
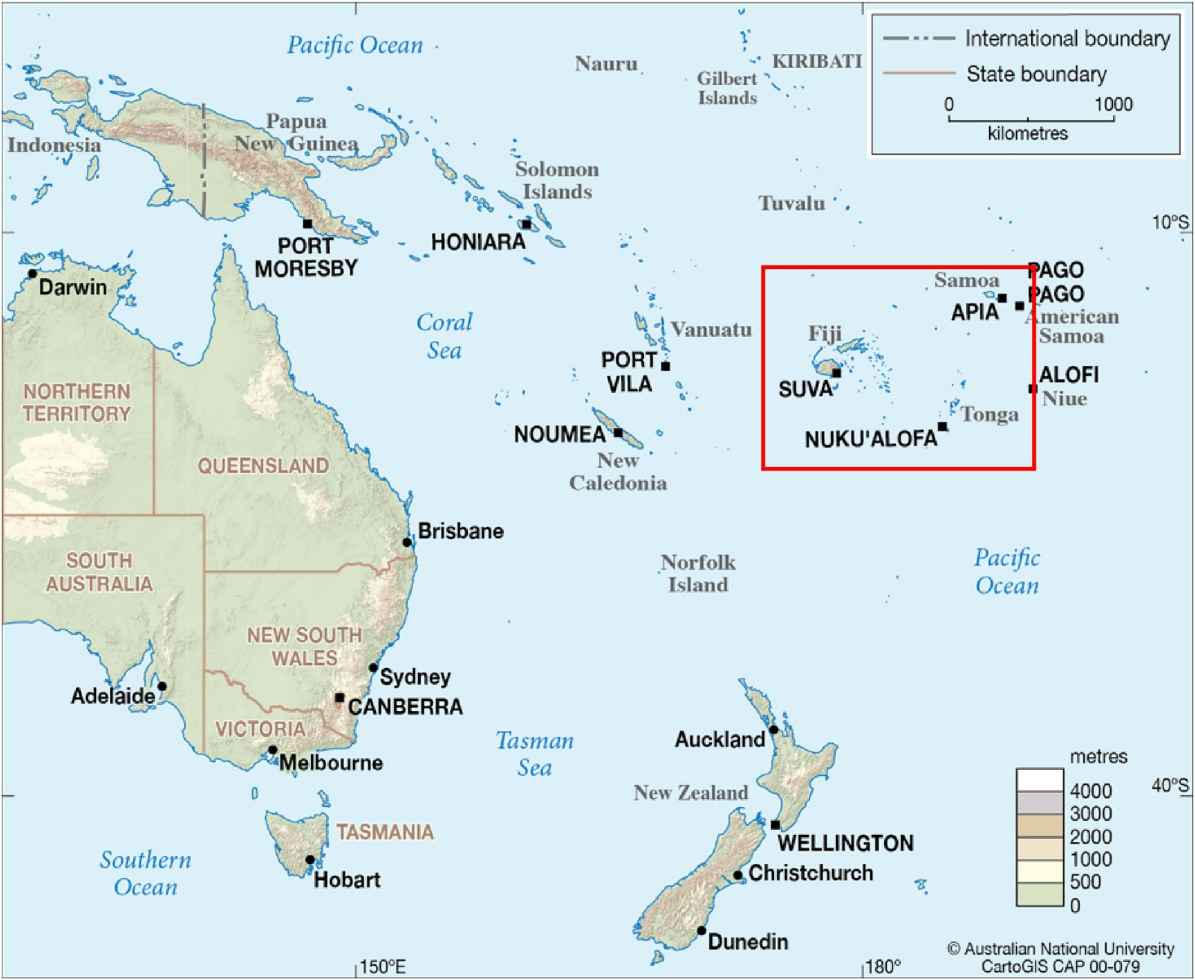
Map of Australia and Pacific region, showing the location of Tonga, Samoa, and Fiji (Map source: CartoGIS Services, College of Asia and the Pacific, The Australian National University 2019)

The Kingdom of Tonga is a Polynesian archipelago of 169 islands located in the Southern Pacific (Fig. 1). Tonga has a population of just over 100,000 people, 70% of which reside on the main island of Tongatapu (Tonga Statistics Department, 2021). Samoa consists of two islands and a collection of small outer islands, with a total population of 200,000, 99% of whom reside on the islands of Upolu and Savai’i. Fiji is an archipelago of 330 islands, with a total population of 880,000, 87% of the population reside on the main islands of Viti Levu and Vanua Levu [18].

**Fig. 2:**
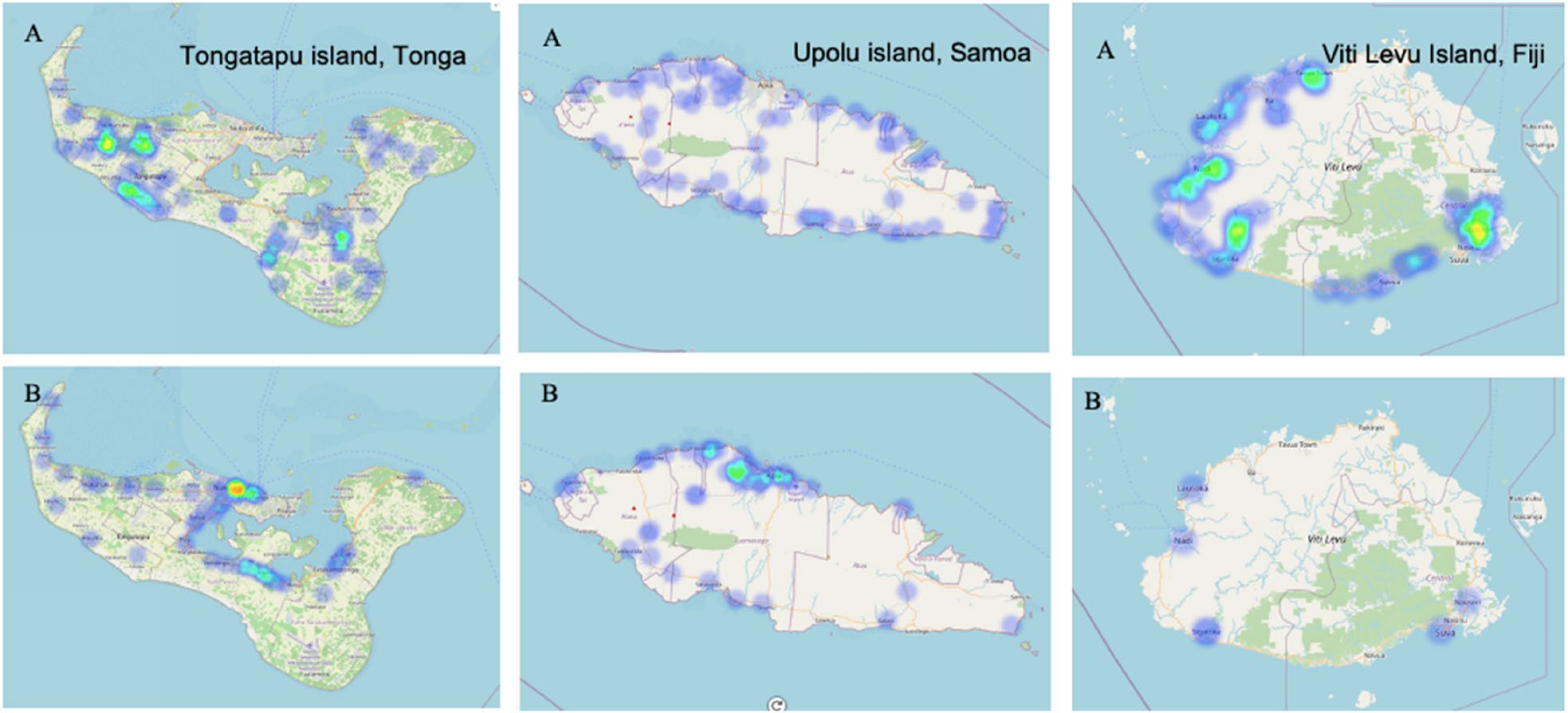
Heat maps illustrating the locations where the farmers (**A**) and market vendors (**B**) were interviewed in Tonga (Tongatapu Island), Samoa (Upolu Island), and Fiji (Viti Levu). The islands shown represent the main and most populated islands within each of the respective island groups

Farmer and vendor surveys were undertaken on Tongatapu Island and the outer islands of ‘Eua and Vava’u (Tonga), Viti Levu and Vanua Levu Islands (Fiji), and Upolu and Savai’i islands, (Samoa). The location (heat map) where farmer and vendor surveys were undertaken on each of main islands in Tonga, Samoa, and Fiji is shown in Fig. 2.

### Data collection

Surveys were undertaken over a five-month period, July to November 2020, which represented the initial phase on COVID-19 restrictions in the region. In Tonga, surveys were undertaken between 2nd July and 24th September 2020; in Fiji between 9th July and July 30th 2020; and in Samoa between 20th July and October 19th, 2020.

### Survey design

Two separate surveys were undertaken, with questions tailored to smallholder farmers or road-side and municipal market vendors. Farmer surveys occurred at the farmer’s place of residence or on-farm. In Tonga and Samoa, market vendor surveys were undertaken at the municipal fruit and vegetable markets or at road-side vendor stalls located throughout each island. In Fiji, vendor interviews were limited to the seven main municipal markets (Nausori, Suva, Sigatoka, Nadi, Lautoka, Labasa, and Savusavu), with Fiji road-side markets not included due to local travel restrictions.

Surveys were semi-structured and involved face to face interviews undertaken in the local language using trained enumerators in each country. Each interview took 10 to 15 min and involved up to 30 questions covering participant’s gender, age, location, farmer or market vendor practice, transport, market accessibility, consumer purchasing behaviour, and postharvest loss during COVID-19 disruptions to the local fresh food system. Interview responses were recorded in English on a tablet or mobile phone with geographic information system mapping (GIS) location capacity using KoboToolBox survey software™ (Harvard Humanitarian Initiative, Cambridge, USA).

### Participants

A total of 825 individuals were interviewed. The socio-demographics and location of those interviewed is reported in Tables 1 and 2, respectively. All participants were randomly selected by local enumerators. Only respondents who confirmed that they were 18 years and older were interviewed, with all interviews completed in compliance with approval from the University of the Sunshine Coast, Australia, Human Research Ethics Committee (A201397), and Fiji Human Health Research and Ethics (FNHHRERC: 09/2020).

**Table 1:** Socio-demographics of farmers and market vendors interviewed

Socio-demographics	Category	Tonga *n* = 260	Fiji *n* = 365	Samoa *n* = 200
Farmers				
Gender (%)	Male	90.1	90	54.5
	Female	9.9	10	43.6
	Not stated	0	0	1
Age (years)	18–30	2.1	10	12.9
	31–50	46.5	54.7	41.6
	51–70	45.8	30.7	40
	> 71	5.6	4.7	3.0
Vendors				
Gender (%)	Male	22.2	34.3	34
	Female	65.2	65.7	66
	Not stated	12.6	0	0
Age (years)	18–30	13.6	9.7	9
	31–50	56.8	40.3	58
	51–70	28.0	45.4	29
	> 71	1.7	4.2 ^a^	3

^a^ 0.5% of participants were not prepared to disclose their age range

**Table 2:** The location where participants were interviewed

Island location	Total number of individuals interviewed ^a^	Number of Farmers	Number of Vendors
Tonga			
Tongatapu Island	176	93	83
Vava’u Island	50	28	22
‘Eua Island	34	21	13
Fiji			
Viti Levu Island	300	120	180
Vanua Levu Island	65	30	35
Samoa			
Upolu Island	140	70	70
Savai’i Island	60	30	30
Total	825	392	433

^a^ Not all participants were prepared to disclose their island location

## Results

### Smallholder farmers

Farmers in Fiji (86%) were more likely to experience difficulties in selling their crops during the initial stages of COVID-19 restrictions, compared to farmers on the smaller Pacific Island nations of Tonga (10%) or Samoa (53%) (Table 3). Fiji farmers reported a reduction in consumer demand, fewer vendors sourcing less product, and that the fruit and vegetable markets were either closed or more difficult or expensive to access. A small cohort of farmers in Fiji (10%), in the regional towns of Tavua, Ba, and Lautoka (Viti Levu Island), and in Labasa (Vanua Levu Island) reported more vendors or that vendors were purchasing more product. Increased vendor participation in these centres might reflect opportunistic trading associated with home gardens, greater local demand due to consumers possibly avoiding the main population centres, or farmers redirecting supply away from the larger municipal markets into the smaller regional towns.

**Table 3:** COVID-19 impacts on smallholder farmer capacity to sell crops in Tonga, Fiji, and Samoa

Interview questions	Farmer response (%)		
	Tonga	Fiji	Samoa
Has COVID-19 impacted on farmers’ ability to sell crops?			
No	37.3	13.3	44.5
Yes	9.9	86.0^a^	52.5
Not sure/no response	52.8	0	4.0
How has COVID-19 impacted on farmers’ ability to sell crops ^b^			
Less consumer demand	0	52.7	18.8
Fewer market vendors	18.3	31.3	7.9
Vendors are buying less product from farmers	16.9	46.7	16.8
Markets or shops I sell to have been closed	3	15.3	13.8
More difficult to access transport to get crops to market	3	8.7	14.9
Transport costs have increased	3	7.3	0
I need to retain more crops for home and village use	0	4.7	0
There is more competition	0	0	22.8
More market vendors	9.2	4.7	17.8
Vendors are buying more product from farmers	4.2	6.7	16.8
Vendors are now selling different type of crops to what I grow	8.5	10.0	23.8
Has crop loss or waste on-farm increased during COVID-19?			
Yes	27.5	72.7	33.7
No	32.4	26.0	63.4
Not sure/no response	40.1	1.3	2

^a^ In Fiji, 42.0% of farmers indicated they were growing less crops; whereas 32.7% indicated they were growing more crops

^b^ Accumulative values that exceed 100% reflect multiple response options. Tonga *n* = 142; Fiji n = 150; Samoa *n* = 99

**Fig. 3:**
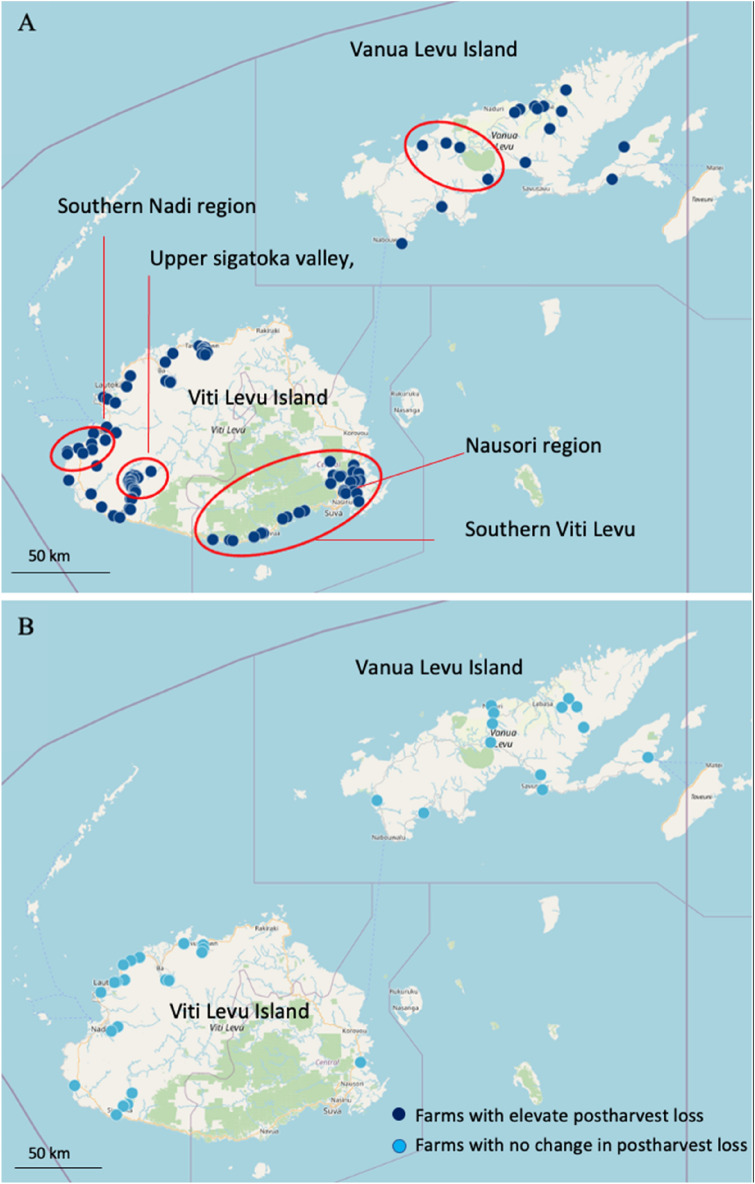
**A** Location of farms on Fiji with elevated horticultural postharvest loss due to COVID-19 impact, **B** farms with no change in their level of horticultural postharvest loss

Most farmers in Fiji (73%) experienced increased postharvest loss due to COVID-19 restrictions (Table 3), consistent with prior reports in the region [8]. Elevated postharvest loss was more prevalent amongst male farmers and farms on the main island of Viti Levu (Table 4), specifically those in the Nausori region, a peri-urban production centre supplying the large Nausori and Suva municipal markets; the lower delta region of Nadi and Southern parts of Viti Levu Island between the villages of Culanuku to Kalokolevu, both key transport routes between Nadi and the capital Suva; and the upper Sigatoka valley, a relatively remote part of one of Fiji’s main horticultural production regions (Fig. 3A, B). Farmers with elevated postharvest loss were more likely to experience difficulties in sourcing critical farm inputs, such as farm chemicals and farm equipment, and consistently highlighted reduced consumer demand, fewer vendors and reduced vendor sourcing (Table 4). The type of market where product was sold (municipal markets, road-side vendor, or direct trade) or the type of crop grown did not appear to contribute to an elevated risk of postharvest loss.

**Table 4:** Farmers in Tonga, Fiji and Samoa with elevated postharvest loss due to COVID-19

Characteristic	Tonga		Fiji		Samoa	
	Increased postharvest loss (%)	No change (%)	Increased postharvest loss (%)	No change (%)	Increased postharvest loss (%)	No change (%)
Farm location						
Main island	45.5	54.5	78.3	21.7	38.8	61.1
Outer islands	47.4	52.6	56.7	43.3	23.3	76.7
Gender						
Female	40.0	60.0	66.7	33.3	32.3	67.6
Male	46.7	53.3	74.8	25.1	50.8	49.2
Farming experience						
Less than 1 year	n/a	n/a	n/a	n/a	21.8	78.1
More than 1 year	n/a	n/a	n/a	n/a	14.8	85.2
Market where product normal sold						
Traders/middlemen	0	0	75.0	25.0	0	0
Municipal market	52.0	48.0	73.5	26.5	41.9	58.1
Road-side vendor	49.2	50.8	72.4	24.1	40.0	60.0
Supermarket or shop			0	0	35.5	64.5
Other (direct supply)^a^	53.1	46.9	73.1	26.9	38.7	61.3
Crop type grown						
Root crops	46.9	53.0	70.1	29.9	34.7	65.3
Fruits	50.0	50.0	70.9	29.1	35.8	64.2
Vegetables	50.0	50.0	73.1	26.9	24.5	65.5
Capacity to produce or sell crops						
Access to farm chemical	81.5	18.5	86.4	13.6	42.1	57.9
Access to farm equipment	90.0	10.0	88.2	11.8	38.5	53.8
Less consumer demand	n/a	n/a	84.8	15.1	61.1	38.9
Fewer market vendors	n/a	n/a	83.0	17.0	75.0	25.0
Vendors are buying less	n/a	n/a	91.4	8.6	52.9	47.1
Access transport	n/a	n/a	83.3	16.7	42.9	57.1
Market closed	n/a	n/a	72.7	27.3	n/a	n/a

n/a. Insufficient number interview responses to analyse or not included interview

^a^ Direct supply to friends, church, hotel, processor, exporter, or not stated

**Fig. 4:**
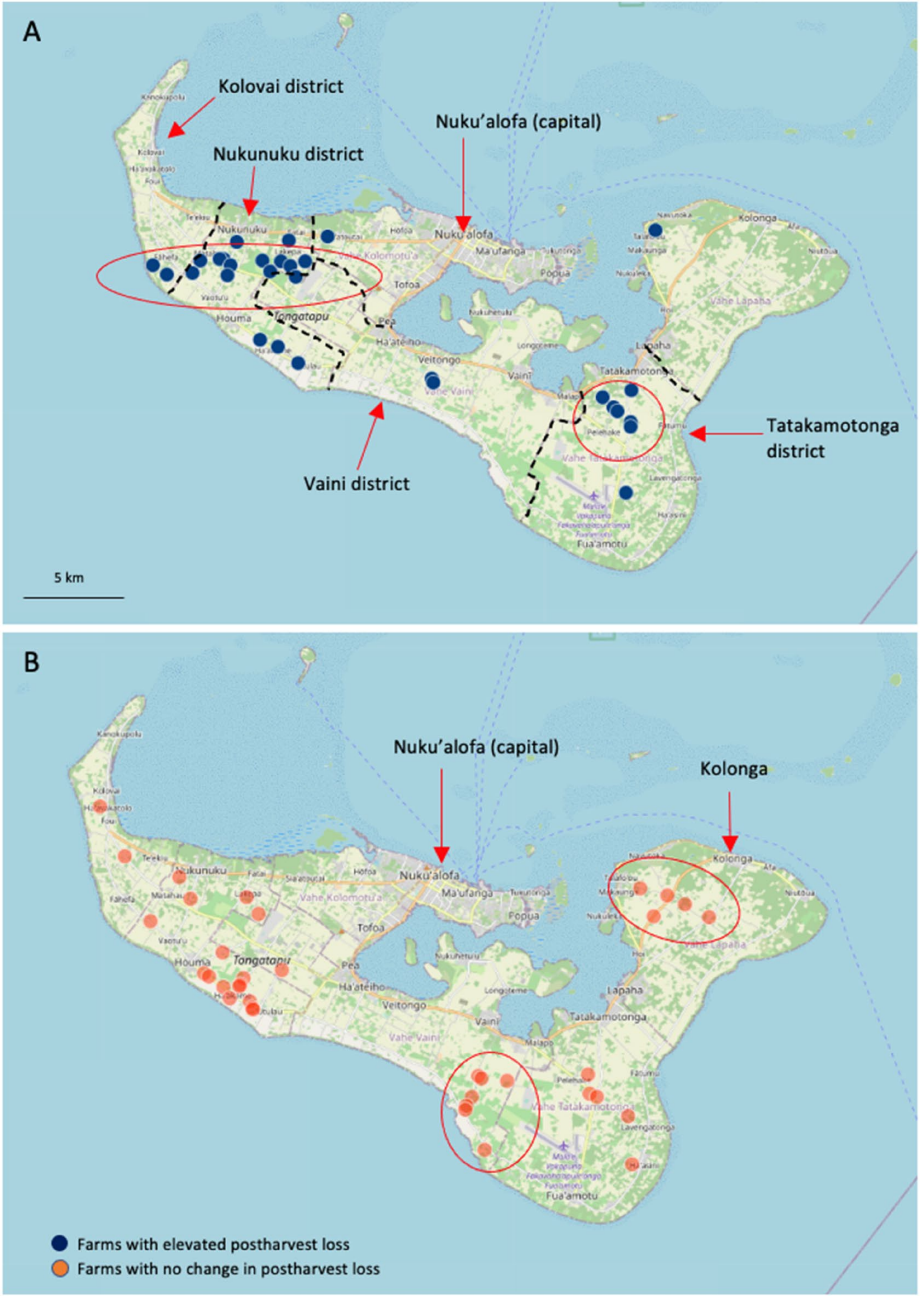
**A** Location of farms on the main island of Tongatapu, Tonga with elevated horticultural postharvest loss due to COVID-19 impact, **B** farms with no change in their level of horticultural postharvest loss

COVID-19 had limited impact on farmers in Tonga, and most farmers did not experience difficulties in selling their crop (37%) or were unsure (53%) (Table 3). The limited number of farmers (10%) who were adversely impacted, mentioned fewer vendors, and reduced vendor purchasing. A small cohort of farmers located in the western district of Nukunuku, Tongatapu Island, and in the eastern parts of Vava’u Island alternatively highlighted that there were more vendors or increased vendor purchasing. Given these locations lack any municipal markets, this is likely to reflect increased road-side vendor trade in the rural areas. Few farmers in Tonga (28%) reported increased postharvest loss (Table 4). Farmers with elevated postharvest loss were located throughout the Tonga island group; however, there was a small cluster of impacted farms in Nukunuku and Tatakamotonga districts, Tongatapu Island (Fig. 4A, B). Gender, market type, and crop type were not associated elevated postharvest loss; however, most farmers who reported elevated postharvest loss also experienced difficulties sourcing farm chemicals and farming equipment. It is unclear why a large cohort of farmers in Tonga were unsure or unwilling to comment on elevated postharvest loss. Given horticultural value chains in Tonga are relatively short with few intermediaries, it would be reasonable to assume elevated postharvest loss would have been detected (Table 4).

Relatively equal proportions of farmers in Samoa reported COVID-19-related impacts on their ability to sell their crop (53%) compared to farmers (45%) that reported no impact (Table 3). Farmers located in rural and remote areas, specifically southern Upolu Island and western Savai’i Island were more likely to report increased vendor number and sourcing, whereas those farmers in northern Upolu Island, including main access roads into the capital Apia, reported fewer vendors sourcing less product.

**Fig. 5:**
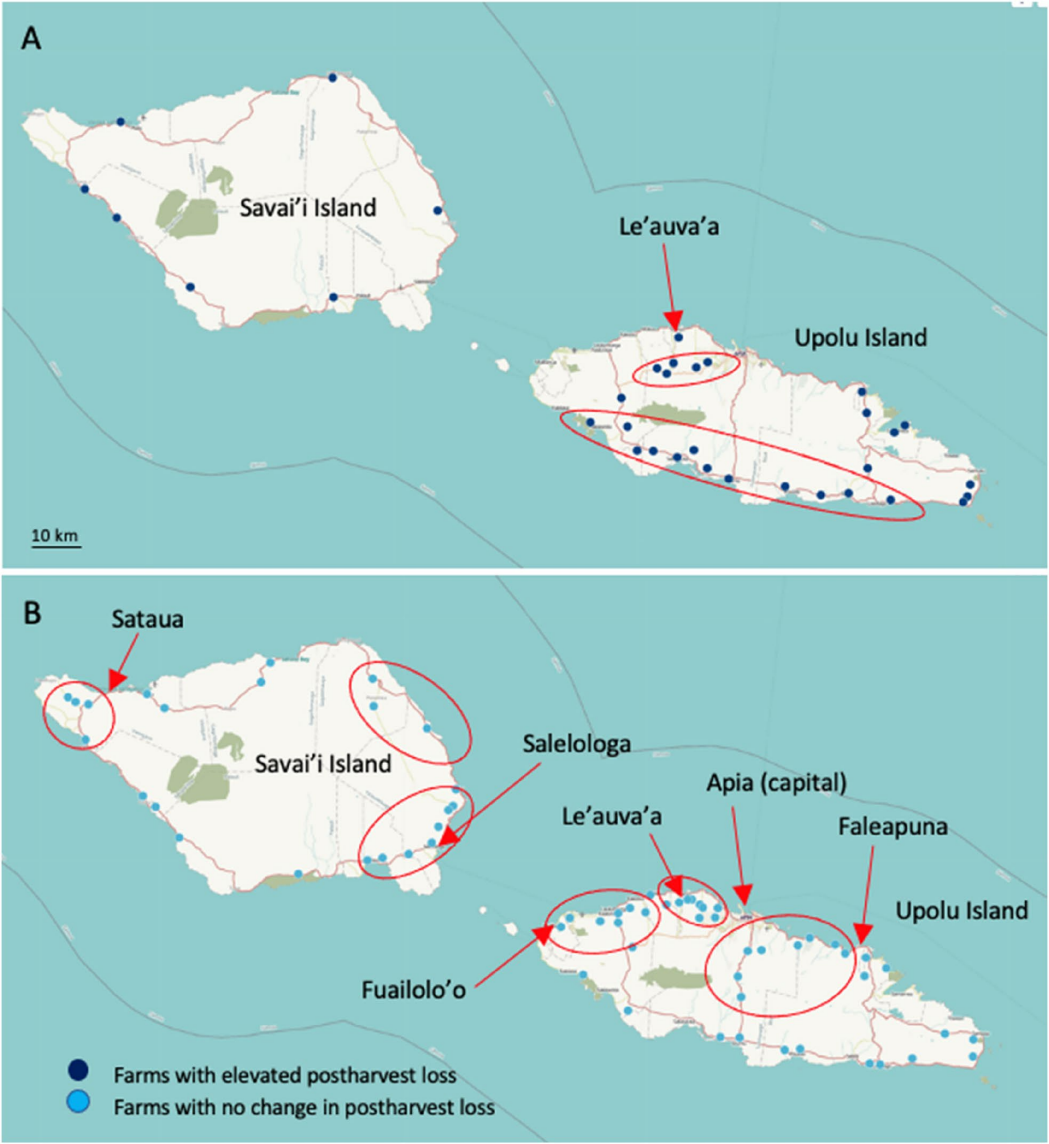
**A** Location of farms on Samoa with elevated horticultural postharvest loss due to COVID-19 impact, **B** farms with no change in their level of horticultural postharvest loss

Few Samoan farmers (34%) reported an elevated postharvest loss due to COVID-19 impacts, consistent with the farmers in Tonga (Table 4). Those farmers who did report elevated loss tended to be male, with farms located throughout the southern parts of Upolu Island, the inland region of Upolu Island adjacent to Le’auva’a, and intermittently along the coastal road around Savai’i Island (Fig. 5A). Farms that did not experience increased loss tended to be more prevalent on north-western parts of Upolu Island between Apia and Fuailolo’o, north-eastern Upolu between Apia and Faleapuna, along the cross-island road (Upolu Island), North-west Savai’i Island near Sataua, and the areas surrounding Salelologa, Savai’i Island (Fig. 5B). Farmers with less than one-year experience were more likely to incur elevated postharvest loss.

### Market vendor

Most market vendors in Fiji (73%) reported that COVID-19 had impacted their capacity to source product (Table 5). Vendors indicated that the cost to purchase fruits, vegetables, and root crops from farmers or traders had increased, that they had less money available to buy product, and that their capacity to source product had been adversely impacted by local travel restrictions. A small percentage of vendors further indicated problems in sourcing product due to farmers harvesting less crops, reduced market trading hours or market closures, and increased transport costs. Despite these challenges most vendors in Fiji (88%) had not altered where they sourced product, suggesting pre-existing supply chain networks had remained relatively intact (Table 5).

**Table 5:** COVID-19 impacts on market vendors in Tonga, Fiji, and Samoa

Participant survey responses (%)	Tonga	Fiji	Samoa
Vendor type			
Municipal market vendors	26.9	100	26
Road-side market vendors	73.1^a^	0 ^b^	70
Permanent vendor stalls			51
Temporary or mobile vendor stalls			19
Retail/supermarket			4
Level of vendor experience (time trading)			
Less than 6 months ^c^	19.5	9.3	17
6 months to 20 years	78	70.4	70
More than 20 years	2.5	20.3	13
Has COVID-19 impacted on your ability to source crops to sell?			
No	43.2	25.5	52
Yes	56.8	73.2	22
Unsure			26
How has COVID-19 impact on your ability to source crops?			
Prices have gone up	11.9	49.5	15
Less money to buy from farmers	7.6	32.9	26
Travel restrictions	31.4	31.9	4
Farmers are harvesting less	16.1	13.4	8
Municipal market was closed or had restricted opening hours	0.8	10.2	22
Transport costs have increased	5.9	7.4	3
Has COVID-19 changed where you source crops from?			
No	90.7 ^d^	87.5	66
Yes	8.5	11.6	8
Unsure			26
Where are you alternatively sourcing product due to COVID-19?			
Wherever I can get a good price	4.2	3.2	–
Traders or agro-marketing agents	0.8	3.2	–
Family farm	0.8	1.4	6
Other market vendors	2.5	0.5	3
Shop or retail outlet	–	–	2
Has consumer demand changed?			
No	15.2	3.2	7
Yes. Consumers are buying less	60.2	58.3	59
Yes. There are fewer consumers	33.1	35.6	61
Yes. There are more customers	16.9	1.9	27
How else has COVID-19 impacted on you as a market vendor?			
I am making less money	65.3	96.8	65
I am making more money	12.7	3.7	20
I am working longer hours	3.4	15.7	19
I am working less hours	27.9	14.4	51
Fewer places to sell (market closed or reduced trading hours)	4.2	6.5	6.0
Has crop loss or waste on-farm increased during COVID-19?			
No	32.2	25.5	55
Yes	67.8	72.2	44
Unsure		2.3	1

^a^ Vendor interviews in Tonga did not segregate road-side vendors according to vendor stall type (trading structures)

^b^ Road-side market vendors were not surveyed in Fiji

^c^ Vendor who commenced trading during COVID-19. The vast majority being road-side vendors (94.1% in Samoa and 82.6% in Tonga). Road-side vendors were not assessed in Fiji

^d^ 79.3% of the vendors interviewed in Tonga sourced product from their own farm or family farm, with 7.4% sourcing product from other vendors (municipal and road-side) and 4.4% from commercial farms

Nearly all Fiji market vendors (97%) were making less money due to COVID-19 impacts (Table 5). Given 67% of vendors interviewed in Fiji were women (Table 1), this represents a disproportional impact on women. Vendors indicated that consumers were buying less product and that there were fewer consumers. In response, market vendors were either working longer hours, possibly to increase net sales, or less hours likely due to a combination of local travel restrictions, reduced market trading hours, and/or a level of disengagement from the market due to reduced profit margins.

A total of 72% of the market vendors in Fiji reported elevated postharvest loss due to COVID-19 impacts, a result consistent with the Fiji farmers response (73%) (Table 5). While increased postharvest loss was reported in all seven of the Fiji municipal markets assessed, it was more prevalent amongst vendors on Vanua Levu Island, female vendors, and those vendors sourcing product from commercial farms (Table 6). The level of market experience had little impact on the likelihood of vendors experiencing elevated postharvest loss. Vendors with less than one-year market experience had similar elevated postharvest loss to those vendors with 11 to 20 years of market experience. Interestingly, the small cohort of vendors with greater than 20 years of experience were more likely to report elevate postharvest loss, (Table 6). It is unclear why this vendor cohort were more vulnerable to elevated loss, given little notable difference in market location, gender, crop type, or product sourcing.

**Table 6:** Market vendors in Tonga, Fiji, and Samoa who reported increased postharvest loss due to COVID-19 verses those who did not experience elevated loss

Vendor characteristics	Tonga		Fiji		Samoa	
	Increased postharvest loss (%)	No change (%)	Increased postharvest loss (%)	No change (%)	Increased postharvest loss (%)	No change (%)
Vendor type						
Municipal market	80.0	20.0	72.2	25.5	76.9	26.1
Road-side vendor (all types) ^a^	62.7	37.3	0^a^	0^a^	34.3	65.7
Temporary vendor stalls					26.3	73.7
Permanent vendor stalls					37.3	62.7
Vendor location						
Main Island ^b^	74.7	25.3	70.6	29.4	61.4	38.6
Outer Islands	51.4	48.6	93.9	6.1	6.7	93.3
Gender						
Female	72.7	27.3	81.0	19.0	43.9	56.1
Male	53.3	46.7	62.1	37.8	47.1	52.9
Vendor experience (years trading)						
Less than 1 year	61.7	38.3	70.8	29.2	32.0	68.0
1 to 2 years (1 to 5 years Samoa only)	76.0	24.0	75.0	25.0	40.6	59.4
3 to 10 years (6 to 10 years Samoa only)	68.6	31.4	0	0	54.2	45.8
11 to 20 years	91.7	8.3	69.8	30.3	n/a	n/a
More than 20 years	n/a	n/a	83.7	16.3	69.2	30.8
Crop type sold ^c^						
Root crops	59.7	40.3	70.8	29.2	42.7	57.3
Fruits	75.9	24.1	75.2	24.8	48.8	51.2
Vegetables	72.2	27.8	76.4	24.6	47.4	52.6
Where is product sourced from?						
Other market vendors	75	25	75.0	25.0	n/a	n/a
Family-owned farm	73.1	26.9	63.5	36.5	40.7	59.3
Trader/middlemen	0	0	61.9	38.1	0	0
Commercial farm	83.3	16.7	81.8	18.2	64.7	35.3
Other ^d^	57.1	42.9	40.0	60	78.6	21.4

n/a. Insufficient sample number

^a^ Vendor interviews in Tonga did not segregate road-side vendors according to vendor stalls (trading structures). Only municipal market vendors were interviewed in Fiji

^b^ Main Island is defined as location where majority of the population reside

^c^ Most vendors sold multiple crop types and multiple products

^d^ Other. Product was sourced from friends or relatives, retail shops, church groups, or importers

The type of crops sold (root crops, fruits, or vegetables) by Fiji vendors did not appear to be a risk factor associated with potential elevated postharvest loss (Table 6). However, most Pacific market and road-side vendors tend to sell multiple products and crop types. When Fiji vendors who only sold vegetables were assessed separately, 75.8% experienced elevated loss (Table 6).

In Tonga, 57% of the market vendors indicated that COVID-19 had impacted on their capacity to sell product (Table 5). By comparison, only 10% of farmers in Tonga were impacted (Table 3). It would appear, COVID-19 impacts on the horticultural systems in Tonga were concentrated at the market end of the chain. Vendors reported local travel restrictions and increased prices to source product from farmers possibly due to farmers harvesting less product (Table 5). Most vendors in Tonga highlighted changes in consumer purchasing behaviour, with 60% of vendors reporting consumers were buying less product, and 33% indicating there were fewer consumers. Nearly all (97%) of the market vendors in Tonga interviewed were making less money (Table 5). Vendors were working less hours possibly due to challenges in sourcing sufficient product from farmers, or a deliberate reduction in the quantity of product sold due to declined consumer demand, local travel restrictions, and a level of disengagement from the market due to reduced profitability (Table 5). A small cohort of vendors (17%), in Tongatapu and Vava’u islands reported there were more consumers, and that vendors were making more money. These vendors tended to represent two distinct cohorts, market vendors who had only recently become active in the market possibly reflecting opportunistic market trading or professional market vendors commonly selling high-value imported product likely to be in limited supply.

Most vendors in Tonga (91%) had not altered where they were sourcing product, consistent with vendors in Fiji (Table 5); however, a significant portion of vendors (19.5%) had only commenced trade since the onset of COVID-19. Given 80% of the vendors in Tonga source product from their own farm, with only 12% of vendors sourcing from commercial farms and other vendors, this result was not unexpected. This high level of family connectivity between farmers and vendors also explains why few vendors in Tonga experienced difficulties purchasing product from farmers, compared to vendors in Fiji and Samoa (Table 5).

**Fig. 6:**
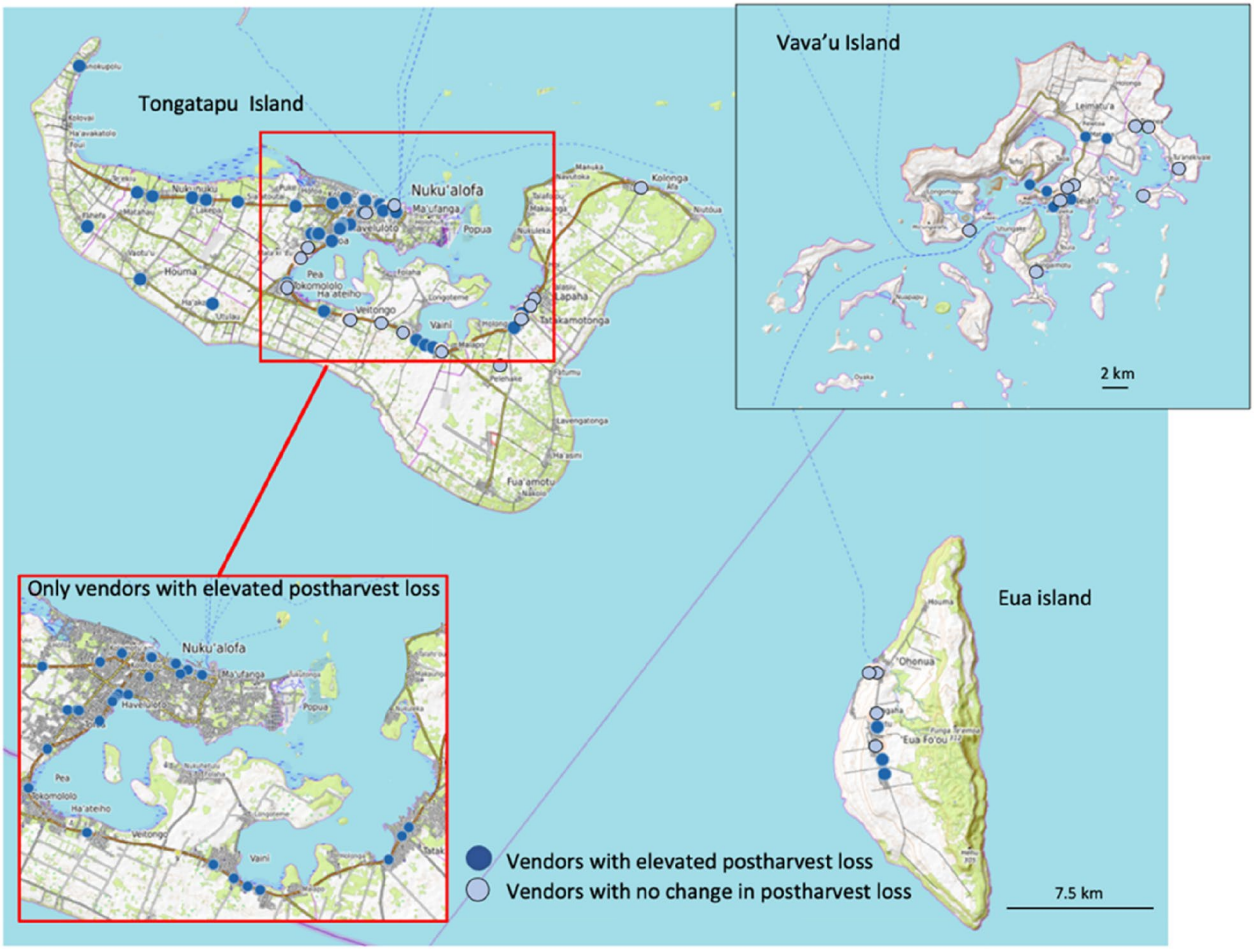
The location of vendors in Tonga with elevated horticultural postharvest loss due to COVID-19, and vendors with no change in their level of postharvest loss

While increased vendor postharvest loss was evident throughout the Tongan archipelago (Fig. 6), it was more prevalent amongst female vendors, those vendors located on the main island of Tongatapu, and vendors in the municipal markets (Table 6). Road-side vendors with elevated loss tended to be concentrated in the western part of Tongatapu Island (Nukunuku and Kolovai districts) and throughout the urban centre of Nuku’alofa (Fig. 6). Conversely, vendors who did not experience increased postharvest loss were located along the main eastern access road between Nuku’alofa and Tatakamotonga, incorporating the districts of Vaini and Tatakamotonga. These village locations and districts are shown on Fig. 4A. This spatial pattern is thought to reflect population demographics, with Nukunuku and Kolovai districts having around half the population, compared to the central and eastern districts Vaini and Tatakamotonga. On the far northern island of Vava’u Island, vendors with elevated loss were more frequently located in the main town of Neiafu, with vendors in the outer rural villages less likely to experience elevated loss (Fig. 6). Collectively, consumers in Tonga appear to be preferentially sourcing product from road-side vendors located close to the main rural population centres, with vendors in the urban centres more likely to experience elevated loss primarily due to reduced consumer demand necessitating prolonged crop storage.

**Fig. 7:**
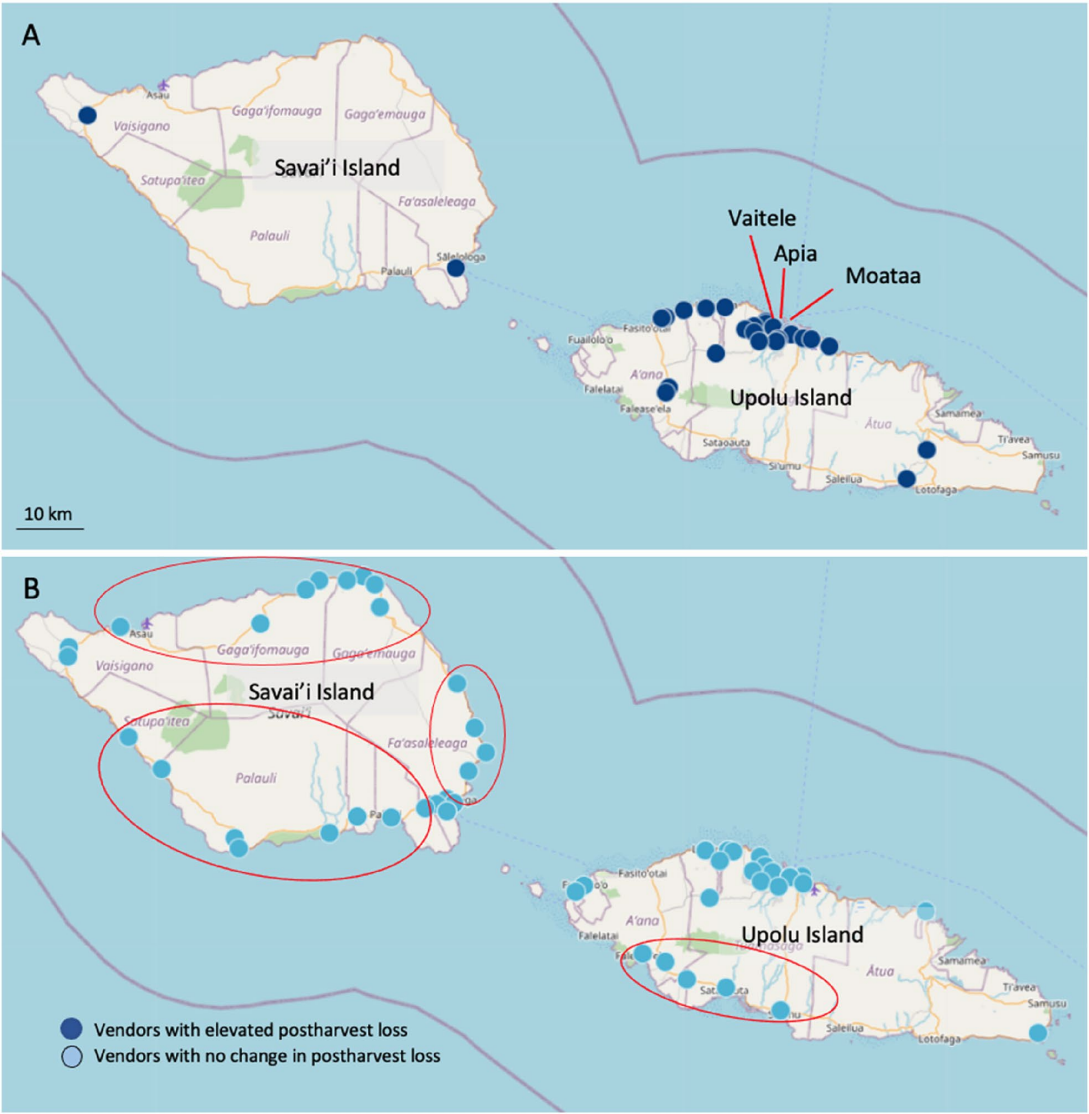
**A** location of vendors in Samoa with elevated horticultural postharvest loss due to COVID-19, **B** vendors with no change in their level of postharvest loss

Tongan vendors with 11 to 20 years of experience were more vulnerable to elevated loss (Table 6). The reason for an elevated risk of postharvest loss amongst the more experienced vendors in Tonga is unclear.

Compared to vendor responses in Tonga (57%) and Fiji (73%), COVID-19 appears to have had less impact on Samoan vendors (Table 5). Only 22% of the vendors interviewed in Samoa indicated that COVID-19 had impacted on their capacity to sell product. Those vendors who were adversely impacted, tended to be in the central municipal market or major road-side cluster markets in the urban centres of Apia, Vaitele, and Moataa, Upolu Island. These vendors reported financial constraints in sourcing product and challenges associated with market closures or reduced market trading hours (Table 5). Interestingly, a significant number of vendors in Samoa (27%) alternatively reported that there were more consumers and that they were making more money (20%). Increased consumer purchasing was restricted to the road-side vendors located in the rural villages throughout Savaii Island and Western Upolu Island, and a few road-side vendors in peri-urban communities bordering Apia. Consumers in Samoa appear to have been sourcing their fruits and vegetables from rural road-side vendors, rather than from the main municipal and urban road-side markets in the main urban centres. As a result, municipal market in the urban markets, particularly professional vendors sourcing from multiple suppliers experienced significant decline in trade, whereas vendors in the rural villages had either stable or increased sales.

Product sourcing and associated farm supply chain networks appear to have remained intact, with only 8% of vendors in Samoa indicated they had changed product sourcing (Table 5). A significant cohort (26%) of vendors in Samoa were unsure whether COVID-19 had impacted on the capacity to sell or if they had altered product sourcing. This cohort were predominately road-side vendors located throughout Upolu Island who were sourcing from their own farms, many of whom had less than 12 months vendor trading experience, possibly indicative of farmers who had recently established a farm-based road-side stall. This might reflect a level of opportunistic trade, in response to consumers increasingly sourcing product locally, or farmers seeking alternative markets due to municipal market closure and reduced urban vendor demand.

Samoan vendors were less likely to experience elevated postharvest loss (44%) compared to vendors in Tonga (68%) and Fiji (72%) (Table 5). Consistent with COVID-19 vendor impact being more prevalent in the urban centres, most municipal market vendors in Samoa (77%) reported elevated postharvest loss, compared to only 34% of the road-side vendors. Interestingly, temporary road-side vendors, those with the capacity to easily relocate their stall to match consumer demand, were the least likely to report elevated postharvest loss (Table 6). Very few vendors (6%) on the outer island of Savai’i reported elevated loss (Fig. 7). While this might reflect reduced consumer sourcing from the main island of Upolu, road-side vender stalls on Savaii commonly sell very small volumes of product, so the underlying risk of postharvest loss was possibly also low.

Unlike Tonga and Fiji, there was little difference between female and male vendors in terms of their risk of incurring elevated postharvest loss (Table 6). Vendors in Samoa with the greatest experience, based on the number of years trading, were more likely to report elevated loss. This result is thought to reflect vendor trading location. More experienced vendors are possibly more likely to be associated with the central urban municipal markets and permanent road-side markets where elevated loss was more common. A significant portion of vendors surveyed in Samoa (17%) had only commenced trading since the onset of COVID-19. While the type of crop sold was not a risk factor associated with elevated risk postharvest loss, vendors who sourced product from friends or relatives, retail shops, church groups, importers, or commercial farms were more likely to report elevated loss. (Table 6).

## Discussion

COVID-19 impacts on South Pacific fresh horticultural food systems during the early stages of the pandemic were far more acute in Fiji, compared to the smaller Pacific Island nations of Samoa or Tonga. Fiji farmers and market vendors reported a reduction in consumer demand and vendor participation in the markets, higher commodity prices, challenges in accessing critical supply chain inputs (i.e. seed and farm chemicals), and a loss of income, collectively reflecting fresh food system impacts reported in other small island developing states [22, 23] and more globally [24–28]. Tonga appears to have been least impacted, with most farmers unsure as to whether COVID-19 had affected their capacity to sell their crops. In contrast, vendors in Tonga experienced a level of market disruption with fewer consumers and reduced product demand. In Samoa, COVID-19 impacts tended to be relatively spatially divergent, with some farmers and vendors reporting reduced consumer demand, and others increased vendor competition and sales.

While COVID-19 had a significant impact on small island developing states [2, 10, 13–17, 22, 23, 29], this needs to be further qualified in terms of South Pacific fresh horticultural food systems. In Pacific Island nations, such as Fiji and Samoa, which have a relatively large urban or peri-urban population and a greater reliance on centralised food distribution systems (i.e. urban municipal markets and supermarkets), the fresh horticultural food system appears to have experienced significant disruption. In Tonga, which has a predominantly de-centralised fresh horticultural food distribution system involving a network of road-side vendors [12] coupled with high levels of household participation in farming, there was reduced or variable food system impacts. It is important to highlight that the wider socio-economic impacts due to COVID-19 in the South Pacific, in terms of loss of income, urban displacement, movement restriction, and disruption to communities were universal and profound [2–4], and consistent with impacts reported in the Caribbean [9, 22, 30], and Maldives, Mauritius, and the Seychelles [23].

COVID-19 increased farmer and market vendor postharvest horticultural loss in Fiji, Tonga, and Samoa. Increased agricultural loss due to COVID-19 has been previously reported in Fiji and the Solomon Islands [8], as well as in Nigeria [24], Bangladesh [27], Zimbabwe [31], India [32], and the Caribbean [22]. While most of these reports attribute elevated loss to disruptions of agricultural transport logistics, market closures and reduced market accessibility, and changes in consumer purchasing behaviour [22, 27, 28, 31, 32], poor postharvest farmer and vendor practice and limited postharvest infrastructure across much of the South Pacific [19, 20, 33] are also likely to have been important contributors. Interestingly, in the Caribbean the level of farmer education was further identified as a risk factor to elevated postharvest loss associated with COVID-19. Farmers with only primary or secondary school education were more likely to incur higher postharvest loss [22]. Farmer education was not included in the current study and therefore cannot be excluded as a possible additional contributor.

Farmer and market vendor postharvest loss may provide an important diagnostic tool in assessing the potential contributors to COVID-19 impacts on Pacific fresh food systems. Postharvest loss in Pacific horticultural systems is commonly low (< 15%), with elevated loss often symptomatic of sporadic supply chain dysfunction or market disruption [12, 19, 20]. In Fiji and Samoa, farmers on the main populated islands of Viti Levu (Fiji) and Upolu (Samoa) were more likely to report higher levels of postharvest loss associated with COVID-19 impacts. In Fiji, elevated loss due to COVID-19 was more prevalent in peri-urban farms located near the main urban centres and in smallholder farms in the upper Sigatoka valley. There was also a cluster of impacted farmers on Southern Viti Levu, a region commonly referred to as the Coral Coast. In Samoa, farms on southern Upolu Island were more likely to experience elevated loss. While postharvest loss was relatively consistent throughout the Tongan archipelago, there were a small cluster of farms in the Nukunuku and Tatakamotonga districts of Tongatapu Island that experienced elevated loss. To understand why these farm locations experienced increased postharvest loss, we need to concurrently consider the market vendors. We found that vendors in the main urban centres of Nuku’alofa (Tonga), and Apia and Vaitele (Samoa) were also more likely to experienced elevated postharvest loss. The incidence of elevated postharvest loss was particularly prevalent amongst municipal market vendors in Tonga and Samoa. Given the Fiji road-side market vendor network was not included in this study, it is unclear whether a similar situation also occurred in Fiji. We believe that local travel restrictions, reduced municipal market trading hours, and local consumers seeking to avoid populated centres that included the urban municipal markets resulted in reduced municipal market fruit and vegetable trading. With poor on-farm and limited market storage infrastructure in Tonga and Samoa [12, 19], urban municipal market vendors and their supporting farm supply chains were more likely to experience elevated loss due to COVID-19. It is difficult to draw wider comparisons with other small island developing states, as there are limited current information on potential spatial variability due to COVID-19 impacts, particularly at the intra-island level.

Road-side vendors were less likely to incur elevated postharvest loss. Relatively lower levels of postharvest loss amongst road-side vendors might be due to increased vendor trading, with consumers possibly preferentially sourcing product from road-side vendors, rather than from the larger urban municipal markets. Road-side vendors are common throughout rural and peri-urban areas in Samoa and Tonga, as well as on the outer islands, possibly providing increased consumer accessibility. In Samoa, Tonga, and Fiji there is an underlying trend towards increased consumer sourcing from road-side vendors and supermarkets [21], often in response to over-crowded and difficult to access urban municipal markets. While local Pacific municipalities often consider road-side markets as problematic due to traffic congestion, COVID-19 impacts to the food system may have inadvertently highlighted the importance of Pacific road-side markets and the merit of decentralisation of fresh food distribution system.

COVID-19 presents an important opportunity to better understand underlying contributors to fresh food horticultural loss in the South Pacific. To date, few studies have sought to examine COVID-19 impacts on postharvest loss in any detail. Spatial mapping of those farmers and vendors with elevated postharvest loss provides new information on those locations and associated value chains in potentially greatest need of support or remediation. In doing so, this enables more targeted interventions to be developed to reduce future Pacific food loss.

This study had several limitations. We were unable to survey Fiji road-side vendors due to local travel restrictions. The exclusion of Fiji road-side market vendors from the market vendor survey resulted in limited insight on the potential impact of COVID-19 on these vendors. COVID-19 impacts on horticultural postharvest loss was limited to determining whether farmers or vendors experienced elevated loss. While this study provided important new information on COVID-19 impact on horticultural postharvest loss on fresh food loss in the South Pacific, a greater depth of understanding as to the nature and extent of food loss impacts could have been achieved had the amount of loss also been quantified. This would have provided a greater resolution to vendor and farm locations with very high levels of postharvest loss due to COVID-19. Farmer and vendor survey results suggested consumer purchasing behaviour was altered. This study could have been further improved had a subsequent consumer survey also been incorporated, enabling a potential further validation of conclusions and further insight into the possible key drivers of consumer purchasing behaviour. During the early stages of COVID-19, the Fiji and Samoan Governments sought to promote home garden production. Similar interventions also occurred in other small island developing states [22]. While the current study did not seek to explore the impact of Government strategies, the targeted promotion of home gardens in the South Pacific in the context of high levels of dietary-based non-communicable diseases across the region [34, 35] warrants further investigation.

## Conclusions

Horticultural farmers and market vendors in Fiji, Samoa, and Tonga were adversely affected by COVID-19. COVID-19 impacts on Pacific horticultural fresh food systems were consistent with that experienced in other small island developing states and developing countries. While the three Pacific islands assessed all had relatively small populations, COVID-19 impact was highly spatially variable. Farmers and vendors on the main islands of Viti Levu (Fiji), Tongatapu (Tonga), and Upolu (Samoa) were more affected than those on the outer islands. Farmers and vendors located or supplying product into urban centres, particularly those aligned to the central municipal markets were also more likely to be impacted, compared to those in rural locations. Changes in consumer purchasing behaviour, possibly due to local travel restrictions, municipal market and supermarket closures, restricted trading hours, or consumers simply seeking to avoid crowded locations, resulted in preferential sourcing of fruits and vegetables from rural road-side vendors. While central municipal markets have historically been the primary distribution centre for fresh fruits and vegetables in the Pacific, COVID-19 has highlighted the critical importance of a de-centralised food distribution system, particularly during periods of external shock. We believe there is a need for a greater recognition and a better enabling policy environment for road-side vendors and peri-urban community markets in the Pacific.

While farm and vendor innovation in response to COVID-19 was not examined, in other small island developing states such as the Caribbean, there was evidence of increased horticultural trading using online social media platforms [22]. Similar trading of fresh foods using social media has also been recently reported in some Pacific Island nations [36, 37]. The emergence of online trading of horticultural crops in the Pacific provides an important area for investigation. Further work is also required to explore Pacific farmer and vendor coping strategies during the latter stages of COVID-19 restrictions, particularly as the region seeks to rapidly normalise its fresh food horticultural systems.

## Data Availability

Not applicable. Human ethic approval conditions precluded data sharing.
